# Proteomic and bioinformatic discovery of biomarkers for diabetic nephropathy

**DOI:** 10.17179/excli2018-1150

**Published:** 2018-03-26

**Authors:** Chadinee Thippakorn, Nalini Schaduangrat, Chanin Nantasenamat

**Affiliations:** 1 Center for Research and Innovation, Faculty of Medical Technology, Mahidol University, Bangkok 10700, Thailand; 2 Center of Data Mining and Biomedical Informatics, Faculty of Medical Technology, Mahidol University, Bangkok 10700, Thailand

**Keywords:** diabetes, diabetic nephropathy, early biomarkers, proteomics, bioinformatics

## Abstract

Diabetes is associated with numerous metabolic and vascular risk factors that contribute to a high rate of micro-vascular and macro-vascular disorders leading to mortality and morbidity from diabetic complications. In this case, the major cause of death in overall diabetic patients results from diabetic nephropathy (DN) or renal failure. The risk factors and mechanisms that correspond to the development of DN are not fully understood and so far, no specific and sufficient diagnostic biomarkers are currently available other than micro- or macroalbuminuria. Therefore, this review describes current and novel protein biomarkers in the context of DN as well as probable proteins biomarkers associated with pathological processes for the early stage of DN via proteomics data together with bioinformatics. In addition, the mechanisms involved in early development of diabetic vascular disorders and complications resulting from glucose induced oxidative stress will also be explored.

## Introduction

**Figure 1:**
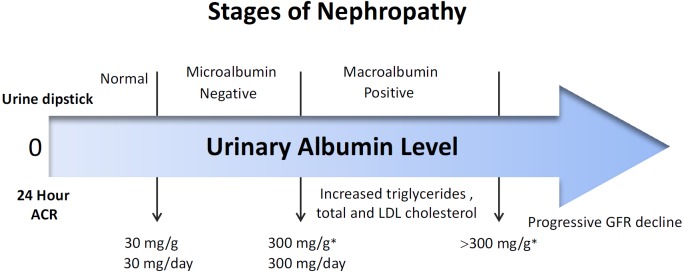
Levels of urinary albumin as measured using various tests at different stages of DN. Thirty mg/day of albumin is regarded to be the upper limit of the normal range for urinary albumin excretion. Conventional urine dipsticks reflect a '+' positive diagnostics at a level of 30 mg/dL albumin that corresponds to 300 mg/L or 300 mg/day. Therefore, a positive value for urine dipstick affords less sensitivity. This indicates a minimal progression of glomerular function.

Diabetes mellitus (DM) is one of the most common chronic diseases associated with abnormally high levels of glucose in blood circulation. The incidence of DM has continuously increased both in the adult and pediatric populations (Ogurtsova et al., 2017[63]). Around 415 million people were estimated to have diabetes in 2015 (Chatterjee et al., 2017[14]), out of which, more than 90 % of individuals had type 2 DM (T2D). In addition, the National Diabetes Statistics Report reveals that up to 9.4 % of the U.S. population had diabetes in 2015, which affected about 30.3 million people of all ages (Gheith et al., 2016[30]). The complications of diabetes such as diabetic nephropathy (DN), neuropathy and retinopathy contribute to increased morbidity and mortality rate of all diabetic patients' worldwide. Moreover, present therapeutic options are still in a dissatisfying condition. Among those complications, DN affects approximately 20-40 % of all diabetics (Molitch et al., 2004[58]). Not only is it the most common cause of chronic kidney disease (CKD), it is also the most frequent cause of end-stage renal failure (ESRD) (Viberti et al., 1982[80]). The earliest diagnostic sign for DN is the presence of detectable amounts of microalbuminuria, indicated with an increase in the level of urine albumin (Figure 1(Fig. 1)) (Mischak et al., 2015[57]). In contrast to type 1 DM (T1D), the levels of microalbuminuria in type 2 DM (T2D) can show fluctuation. This can thus, lead to the development of macroalbuminuria in 20-40 % of diabetic individuals (Molitch et al., 2004[58]). Moreover, a 40-50 % increase of proteinuria has been evidenced to promote the development of chronic renal failure (CRF). In addition, up to 50 % of patients with T2D progress to cardiovascular diseases and eventually death. Therefore, obtaining early diagnosis of individuals with risk for the development of DM complications is extremely important for the prevention of further disease progression (Campion et al., 2017[13]; Kim et al., 2007[41]). However, no specific diagnostic biomarkers for T2D with nephropathy is currently available other than microalbuminuria or macroalbuminuria. Although microalbuminuria (MAU) has been considered to reflect the early stage of the irreversible process of nephropathy, the predictive value of MAU for type 2 diabetics with nephropathy is still insufficient. 

Proteins are responsible for all biological processes and environmental influences. Hence, many biomarkers that were established in the past decade consist of a large number of proteins. Recently, the proteomics technology has been applied in many research fields for the discovery of novel biomarkers that can be useful in discerning certain diseases and their associated complications, including DN. Furthermore, potential biomarkers with the ability to ascertain higher specificity and accuracy for clinical implementation are also sought out through proteomics. Moreover, studies on proteomics are useful for understanding the mechanisms of pathology. Serum proteomics is the most popular target for the investigation of disease related biomarkers due to the containment of numerous protein information (Kim et al., 2007[41]; Vitova et al., 2017[81]). Urinary proteomics is another source used in the identification of non-invasive biomarkers (Devarajan, 2010[19]). Previously, Devarajan reviewed existing CKD biomarkers in an effort to gain an understanding on the complex pathophysiologic processes underlying CKD progression. 

In an extension of this aforementioned work, this review aims to cover the current and novel plasma protein biomarkers in the context of DN in order to assimilate knowledge from the early stage of disease development and identify potential proteomics biomarkers for actual clinical implementation. In addition, this article also reveals the pathophysiological roles of hyperglycemic related renal disease such as oxidative stress induced conditions. It is anticipated that the information gathered herein regarding the mechanism may be useful for the discovery of potential diagnostic and therapeutic targets.

However, major challenges in synthesizing these discoveries and translating them to clinical practices still re-main. In recent years, bioinformatics have been instrumental in the study and analysis of the rapidly increasing proteomics data. Therefore, in this review we also discuss the implementation of bioinformatics and machine learning in their roles on the identification of novel biomarkers of DN.

## Current Biomarkers of Nephropathy Used in Clinical Diagnosis

### Glomerular filtration rate

Glomerular filtration, which is a process where the kidneys remove excess waste and fluids by filtering blood, can be used to estimate the kidney function using the rate of blood filtration. As such, the glomerular filtration rate (GFR) is an assessment of the remaining function of kidneys. GFR is also commonly applied as a biomarker for the prediction of chronic kidney disease stages. However, it should be noted that GFR cannot be measured directly. Measurements are traditionally based on the renal clearance capacity of endogenous biomarkers in plasma, expressed as the volume of plasma completely cleared of the biomarker per unit time. This test, known as the estimated GFR (eGFR), commonly measures the levels of serum creatinine (SCr) or cystatin C (CysC) in the blood and the result is used in combination with age, sex and weight of patients in order to calculate how well kidneys are functioning. Nevertheless, biomarkers used to measure GFR can also be exogenous substances such as inulin, iohexol, iothalamate, technetium diethylenetriamine-pentaacetic acid (TcDTPA), chromium labelled ethylenediaminetetraacetic acid (CrEDTA), etc. 

Although GFR is considered as the most important biomarker of kidney function, the calculations are time consuming and require experienced personnel for its implementation. Moreover, the estimated value from equations can promote some negligible systematic bias in which the average deviation from the assessment of GFR are imprecise, which shows approximately 10-20 % of estimates deviates by more than 30 % from the measured GFR value (Brenner et al., 1978[11]; Levey et al., 2015[47]).

### Microalbuminuria

Microalbumin is an outcome predictor in patients with renal disease, as such; the determination of minimum levels of urinary albumin excretion (microalbuminuria) can be implied for incipient diabetic kidney disease. Microalbuminuria has been identified for the discharge of 30 to 300 mg of albumin per day (or 20 to 200 μg/min or 30 to 300 μg/mg creatinine) in 2 out of 3 urine collections. This phase calls for aggressive management in order to prevent retard overt DN. Additionally, microalbumin can be used as a predictor of morbidity and mortality in patients with no signs of renal diseases. 

In hypertensive patients, microalbuminuria has also been associated with the enlargement and thickening of the left ventricle (left ventricular hypertrophy). Furthermore, for hypertensive and normotensive patients, microalbuminuria can be used as a biomarker for the increasing risk of cardiovascular diseases and early cardiovascular mortality. However, performing a conventional 24-hour urine collection for testing of proteinuria and measurement of creatinine clearance are optional because the measurement of a spot urine collection for the assessment of albuminuria to creatinine ratio is acceptable for diagnostic and therapeutic implementation. Remarkably, exercise, dietary protein intake, vasoconstriction in the upright posture, pregnancy, fever and activation of the renin-angiotensin system tend to increase albumin excretion rates and there is significant diurnal and day-to-day variation (Brenner et al., 1978[11][12]). On a basic diagnostic urine test strip or dipstick, 10 to 20 mg/dL is the minimal detection limit of protein. A positive predictive value for the dipstick therefore, only indicates the possibility of microalbuminuria but it is not an exact form of diagnosis.

### Creatinine

Creatinine is a 113 Da protein, obtained as a by-product from the breakdown of creatine phosphate in muscles. It is completely filtered but not reabsorbed by the glomerulus nor is it metabolized and thus, is excreted unchanged from the kidneys. However, a significant percentage of creatinine in the urine is derived from the proximal tubular secretion. Thus, plasma creatinine levels are produced at a relatively constant rate based on age, gender and muscle mass (0.8 to 1.4 mg/dL in adult males and 0.6 to1.2 mg/dL in adult females). 

A deficient filtration process in the kidney can be indicated with an increase in concentration of blood creatinine levels. Consequently, the level of blood and urinary creatinine can be applied for the determination of the creatinine clearance (CrCl), a biomarker which corresponds to GFR. In clinical laboratory conditions when the estimated GFR based on creatinine is predicted as not accurate enough for clinical decision making, confirmatory tests are needed for exact determination of GFR. These include estimating GFR using serum cystatin C (eGFRcys) or a combination of both (eGFRcr-cys).

### Blood Urea Nitrogen (BUN)

Urea is the primary metabolite derived from dietary protein and tissue protein turnover. It is a relatively small molecule consisting of 60 Da that can be distributed throughout the body via the blood circulation. Therefore, the term of blood urea nitrogen (BUN) refer to the level of urea nitrogen in blood and serum. 

The normal value of BUN is approximately 5 to 20 mg/dL, or an equivalent amount of 1.8 to 7.1 mmol/L. The broad range of this normal condition is acceptable due to individual variations such as dietary protein intake, endogenous protein catabolism or breakdown, hydration state, urea production and metabolism inside the body. However, the indications of BUN levels are non-specific to renal functionality. Several factors are involved in the determination of BUN levels which include blood volume, circular blood flow, high protein diet and also some complications such as febrile illness, gastrointestinal bleeding etc. 

Owing to limitations of current biomarkers of renal failure and DN (i.e. low accuracy and non-specificity of such biomarkers due to variations on age, sex, body weight, protein intake and catabolism; complications arising from factors pertaining to the state of hydration, fever or even pregnancy of patients) there is a substantial need for the discovery of novel serum biomarkers.

## Diabetic Nephropathy and Biomarkers of Glomerular Damage

Diabetic nephropathy (DN) is characterized by damages to kidneys as caused by diabetes. Changes in glomerular permeability and structure are significantly defined as biomarkers for evaluation. Generally, the composition of glomerular capillary wall comprises of endothelial cells, basement membrane and epithelial cells. The selectivity in glomerular membrane filtration is present in the basement membrane, which is a part of the kidney that helps filter waste and extra fluids from the blood. Importantly, proteins that are not filtered, but retained in the circulation is due to their size and charge (Brenner et al., 1978[12]; Matheson et al., 2010[54]). However, damage of the basement membrane or changes in permeability barrier leads to protein exclusion failure, which promote urinary protein excretion. Consequently, proteinuria of plasma proteins (e.g. albumin and transferrin) that are normally not freely filtered through the glomerulus are detectable (Najafian and Mauer, 2009[60]). In addition, structural changes associated with diabetes-related kidney failure includes the increase in glomerular extracellular matrix accumulation and thickening of the basement membrane in the glomeruli (Ziyadeh, 1996[93]), as well as renal tubular hypertrophy and associated basement membrane alterations in the tubulointerstitium with tubolointerstitial fibrosis (Al Hariri et al., 2017[4]). These abnormalities are allied with renal over production of extracellular matrix proteins, such as type IV collagen (Brenner et al., 1978[12]) and other proteins. Diabetic mouse models demonstrated the aorta and kidney responses to the diabetic state differently. Protein expression profiling studies in mouse tissue model revealed the presence of protein modification, which affected a wide variety of protein function such as proteins involved in the inflammatory processes, fibrotic, oxidative, cytoskeleton and their related proteins. Moreover, protein networking and interconnection to vascular disorders and also kidney disease are detectable.

## Oxidative Stress in Diabetic Nephropathy

Chronic hyperglycemia has been reported for a decade as a condition that is vigorously associated with diabetes complications. Long-term damage, dysfunction and chronic failure of various organs (e.g. eyes, kidneys, nerves, heart and blood vessels) (Pu et al., 2006[66]) are the major causes of illness and death in population suffering from diabetes. 

Nowadays, the elucidation of mechanisms associated with DM that promotes their complications, is still elusive due to the complexity of biological processes that might be involved. However, the evidence of direct toxic effects of high blood glucose concentrations on the increase of high blood pressure, abnormal lipid metabolism, oxidative stress, chronic inflammation, hypoxia and ischemia (Evans et al., 2002[24]) have been revealed. 

Investigations suggest that oxidative stress plays a pivotal role in the pathogenesis, progression and complications of T2D (Aghadavod et al., 2016[3]; Giacco and Brownlee, 2010[32]). Systemic effects especially cellular dysfunction as provoked by lipid peroxidation induced-oxidative stress as well as important biomolecule alterations (e.g. protein and DNA) is strongly correlated to elevated amounts of reactive oxygen species (ROS) that is produced in various tissues under diabetic conditions. 

An important outcome of oxidative stress affecting the biological cascade is the abnormalities of metabolism in diabetic individuals and the overproduction of mitochondrial superoxide radicals in endothelial cells lining. The higher production of superoxide radical leads to the stimulation of biological pathways that are involved in the pathogenesis of diabetic complications, such as the polyol pathway flux (the sorbitol-aldose reductase pathway), increased production of advanced glycation end products (AGEs), higher expression of AGEs ligands and receptors, enhancement of the isoforms of protein kinase C, hexosamine pathway activity increase and DNA lesions such as DNA strand breaks and protein crosslink formation as well as oxidative damage. 

Some evidence demonstrates that AGEs play a crucial role in the development and progression of diabetic vascular damage. Accumulation of AGEs in the kidney may contribute to a variety of microvascular and macrovascular complications through the formation of crosslink between the extracellular matrix basement membrane molecules by engaging the receptor for AGEs (RAGE) (Basta et al., 2004[8]). RAGE works as a signal transduction receptor molecule that can bind to S100 calgranulins and amphoterins, which represent non-AGE pro-inflammatory molecules. The activation of multiple signalling pathways is brought about by the accumulated expression of RAGE associated ligands at sites of high tissue damage (Tan et al., 2004[73]). Furthermore, the activation of key transduction pathways by RAGE due to AGEs leads to increased production of pro-inflammatory cytokines. 

**Figure 2:**
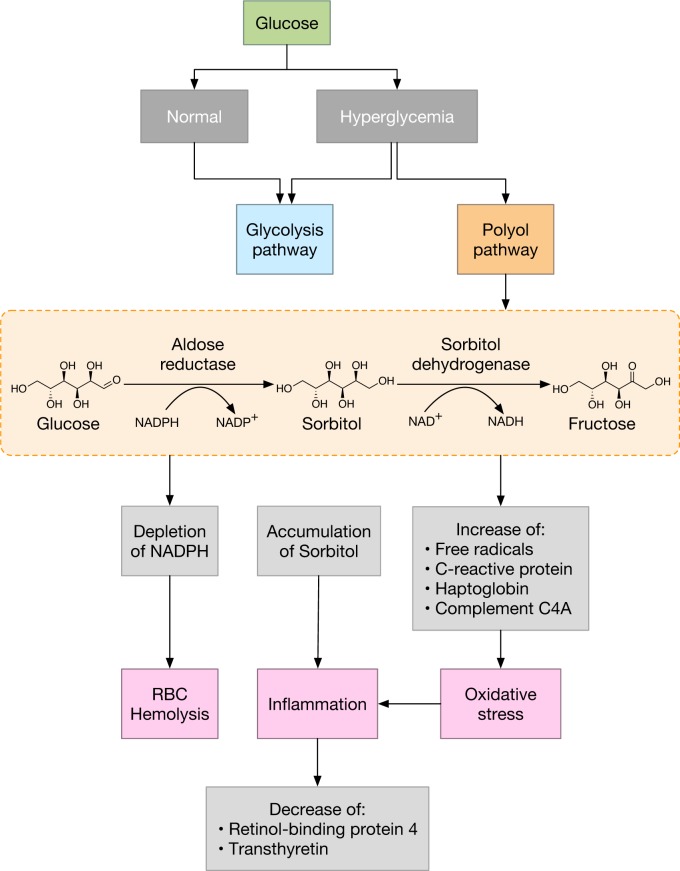
Schematic representation of hyperglycemia induced oxidative cascade in diabetic complications. Under normal conditions, glucose is taken up by the skeletal muscles and adipose tissues and broken down in the glycolysis pathway, providing energy for cells. Excess glucose however, enters the polyol pathway. Hyperglycemia develops when overly excessive amounts of glucose are present. The affinity of aldose reductase for glucose increases, causing sorbitol to accumulate and allowing excess NADPH to be used. This in turn decreases the amount of available NADPH leading to RBC hemolysis and oxidative stress whereby free radicals and acute phase proteins such as C-reactive protein, haptoglobin and complement C4A increase. After RBC hemolysis, free hemoglobin binds to the haptoglobin α2 chain resulting in a complex (Hb-Hp) that is eventually cleared by the CD163 macrophage receptor, resulting in the decrease of the haptoglobin α2 chain. In addition, the accumulation of sorbitol and free radicals leads to cellular inflammation such that, the inflammation of adipose tissues leads to the decrease of retinol-binding protein 4 and transthyretin.

In addition, macrophages are induced to release IL-6, tumor necrosis factor (TNF)-α and IL-1β upon stimulation with AGEs. Thus, the production of C reactive protein (CRP) might be initiated by the aforementioned signalling cascade, which are stimulated by AGEs on macrophages (Pickup et al., 1997[65]). These abnormalities eventually lead to an increase of serum or plasma concentrations of several acute phase proteins such as CRP, serum amyloid A, fibrinogen, α1-acid glycoprotein and plasminogen activator inhibitor-1 (Pickup et al., 1997[65]; Vanizor et al., 2001[78]). Similarly, our results revealed changes in protein expression levels of many acute phase proteins including haptoglobin, fibrinogen, retinal-binding protein 4, complement factors, etc. (Table 1(Tab. 1) and Figure 2(Fig. 2)). 

In addition, hyperglycemia has been found to promote the release of free radicals while reducing antioxidant defense responses, which are correlated to endothelial dysfunction. Therefore, high potential biomarkers of oxidative stress and stress defense responses could indicate the progression of diabetes and its complications (Najafian & Mauer, 2009[60]). Numerous urinary biomarkers related to oxidative stress have been studied and reported, which corresponds to diabetes and its chronic complications including, 8-hydroxy-2'-deoxyguanosine (8-OHdG, or 8-oxodG) and pentosidine (Dronavalli et al., 2008[21]).

## Hyperglycemia at the Early Stage of Diabetic Nephropathy

Hyperglycemia actively promotes the cascades of cytokine and growth factors thereby mediating the damaging effects that enhances the production of oxidative stress that is in concomitant with increases in pro-inflammatory mediators, lipid peroxidation and atypical glycosylation (Ha et al., 2008[35]). Furthermore, there is evidence suggesting that at the early stages of DN, TNF-α can act as a useful biomarker owing to the development of pathological albuminuria being preceded by its high concentrations. 

Nevertheless, further research is needed to discern whether the increase in this biomarker does in fact correspond to the progression of nephropathy. Evidences supporting the utilization of 8-OHdG and pentosidine as oxidative biomarkers for the determination of DN and its related complications exists. However, these biomarkers should be compared to urinary albumin in terms of their specificity and sensitivity (Choudhary and Ahlawat, 2008[17]; Gorin and Wauquier, 2015[33]). 

Studies pertaining to renal injury associated with metabolic disease and their underlying pathogenesis suggests that oxidative stress is triggered by an increase of ROS production, impaired mitochondrial functions and/or incapacitated antioxidant systems. Various areas of ROS exist in the kidneys (e.g. the electron transport chain in the mitochondria, xanthine oxidase and uncoupled nitric oxide (NO) synthase). On the other hand, nicotinamide adenine dinucleotide phosphate (NADPH) oxidase is considered to be the major producer of ROS (Chen et al., 2013[15]). NADPH oxidases are multi-subunit enzyme complexes composed of membrane and cytosolic components that primarily function as electron transporters across cell membranes. 

Seven members are found to make up the NADPH oxidase (Nox) family, which include Nox1-Nox5 and dual oxidases (Duox), Duox1 and Duox2, both of which are found in different tissues and are seen to be activated via different mechanisms (Teng et al., 2014[74]; Wan et al., 2016[82]). Moreover, mechanisms involved in the regulation of Nox activity are homologue specific, which are related to the operation of protein-protein complexes and interactions, protein phosphorylation and protein translocation from one site of the cellular compartment to another as well as the activation of Rac (a subfamily of the Rho family of GTPases). 

The overproduction of reactive oxygen species (ROS) corresponds with the upregulation of Nox protein. Several stimuli and agonists have been observed to activate ROS production including hyperglycemic condition, expression of transforming growth factor-β (TGF-β), and angiotensin II (Ang II), production of oxidized low density lipoprotein (oxLDL), insulin-like growth factor-1 (IGF-1) as well as the release of vascular endothelial growth factor (VEGF) and mineralocorticoid hormone aldosterone, etc. (Teng et al., 2014[74]).

In hyperglycemia, the metabolism of excess glucose takes place via numerous pathways such as the polyol pathway whereby sorbitol is produced from glucose. However, during this process antioxidants such as glutathione (GSH) are drained while ROS levels rise. Furthermore, the formation of AGEs occurs due to excess glucose binding to free amino acids via condensation, which then modulate several important events like the induction of protein kinase C (PKC) (Basta et al., 2004[8]; Ha and Lee, 2005[36]), which then triggers the production of ROS via NADPH oxidase (Larance and Lamond, 2015[45]). 

All of these pathways correlate with one another in such a way that upon an increase of oxidative stress through AGEs and PKC, the further production of AGEs and PKC is also enhanced in a positive feedback cycle. Due to the accumulation of oxidative stress and modified elements, adverse consequences are generated which include the activation of C-reactive proteins and pro-inflammatory cytokines etc.

## Acute Phase Proteins as Early Biomarkers of Diabetic Nephropathy and Other Diabetic Vascular Damage

In order to understand the relationship between serum or plasma proteins in hyperglycemic patients and their related conditions as well as the pathophysiology of such conditions, it is pertinent to acquire increased knowledge regarding early biomarkers for better diagnosis and prevention in the early stage of the disease. Several individual proteins have been described as representatives of novel biomarkers for kidney disease. These biomarkers were accurately identified by means of available immunological assays as well as with the integration of high throughput approaches such as proteomics technology for increasing the chance of novel biomarker discoveries.

Proteomics has extensively been applied not only to discover new biomarkers but also to gain a better understanding on the underlying mechanisms of several diseases (e.g. diabetes, cancer, Alzheimer's and renal disease). In this regard, proteomics based-approaches will be useful due to the power of this technology to detect and identify protein expression levels in cells and biological samples (Mahfouz et al., 2016[52]; Zhang et al., 2013[91]). Protein profiling of diabetes and their related disorders have been extensively studied for more than a decade. For instance, differential expression of retinol binding protein 4 (RBP4) has been observed and found to correlate with the duration of diabetes and DN (Liu et al., 2016[48]). Furthermore, potential protein biomarkers (e.g. haptoglobin, fibrinogen and serum complement factor) have been discovered for diabetic vascular diseases (Asleh and Levy, 2005[5]; Fujita et al., 2013[28]; Yang et al., 2005[89]).

A study conducted by our group on 75 hyperglycemic plasma samples using two-dimensional gel electrophoresis (2-DE) in conjunction with liquid chromatography-mass spectrometry (LC-MS/MS) was successfully carried out in order to investigate changing profiles of protein expression as a consequence of elevated fasting blood glucose in different concentrations (normal group: <100 mg/dL; borderline high group: 100-126 mg/dL; and high group: > 126 mg/dL) with complications of microalbuminuria (>30 mg/dL) and macroalbuminuria (>300 mg/dL) (data not shown). Plasma samples were collected as leftover specimens from the Center of Medical Laboratory Services, Faculty of Medical Technology, Mahidol University, Thailand. 

Briefly, pooled plasma samples were generated for each group and abundant proteins in serum samples (e.g. albumin and immunoglobulin) were removed using the trichloroacetic acid/acetone precipitation method. Total protein in each group was subsequently precipitated and protein expression levels were quantified using 2-DE and LC-MS/MS. Finally, peptides recorded using MS/MS spectra were compared using the MASCOT program against protein sequence databases such as NCBInr and SwissProt. 

Results from our findings indicated that there exists a differential expression level in plasma protein samples when compared to samples with normal glucose level. Changes in protein expression includes the reduction of retinol binding protein, transthyretin and zinc-α-2-glycoprotein levels for the hyperglycemic group while an increase in complement factors C4-A and C3. In addition, acute phase protein, namely haptoglobin, was also detected in hyperglycemics and hyperglycemics with microalbuminuric conditions (Table 1(Tab. 1)). Of all the above mentioned biomarkers, differentially expressed proteins are known to be involved in inflammatory processes particularly those classified as acute phase proteins which play a vital role in glucose metabolism and transportation.

### Retinal binding protein

Retinol binding protein 4 (RBP4) or retinol binding protein (RBP), carries retinol from the liver to the perimeters by acting as a plasma transporter. RBP4 has been reported as a negative acute phase inflammatory reactant. Several evidences reveal the correlation of elevated RBP4 expression in insulin-resistance associated obesity and T2D (Aeberli et al., 2007[2]). Moreover, other studies observed high concentrations of RBP4 in obesity (Zabetian-Targhi et al., 2015[90]) inducing chronic inflammation and in its complications such as T2D, metabolic syndromes and cardiovascular diseases (CVDs) (Codoner-Franch et al., 2013[18]). In addition, RBP4 was seen to positively correlate with GLUT4, (Codoner-Franch et al., 2013[18]) the insulin-regulated glucose transporter found primarily in adipose tissues and striated muscles. Transgenic expression or injections of RBP4 was able to induced insulin resistance in a mouse model, in contrast to the reduction of RBP4 expression with improved insulin resistance in diet-induced obesity (Aeberli et al., 2007[2]). However, much research carried out between the correlation RBP4 and oxidative stress show positive correlations with markers such as urinary 8-isoprostane, 8-isoprostaglandin F2α (8-isoPGF2α), 13-(S)-hydroxyoctadecadienoic acid and malondialdehyde (Ghosh et al., 2015[31]; Liu et al., 2014[49]) while a negative correlation between RBP4 and antioxidant glutathione was also reported (Liu et al., 2014[49]). Thus, RBP4 may have a role in oxidative stress induced diabetic complications. In parallel, transthyretin (TTR) is a transport protein involved in the blood transport of different molecules with high binding capacity for thyroxine (T4), triiodothyronine (T3) and holo-retinol-binding proteins. In the blood circulation, RBP4 forms a complex with TTR, which increases the molecular mass of RBP4 leading to the prevention of glomerular filtration and excretion through the kidneys. These evidences lend support to the positive correlation between the expression levels of RBP4 and TTR.

### Complement factor

The complement system is made up of more than 30 plasma and cell membrane proteins and it acts in both the adaptive and innate immunity in the form of an effector. These factors are produced by hepatocytes. In addition, proteins of the complement system works together via the classical, alternative and mannose-binding lectin pathways, which are enzyme activating pathways. The three activation pathways eventually induce a generation of membrane attack complex (MAC), the main effector of the complement-mediated pathway, leading to tissue damage (Flyvbjerg, 2017[26]). Chronic inflammation is characteristic of T2D. The induction of proinflammatory cytokines is induced from obesity activated adipocytes, which release adipocytokines that eventually leads to impaired vascular endothelial cells and organ injury. C3a is a candidate that can induce tissue inflammation and damage (Fujita et al., 2013[28]; Flyvbjerg, 2017[26]). Moreover, using network analysis of diabetic rats, the relationship between the up-regulation of complement C3 and the activation of NF-κB and TNF was elucidated (Al Hariri et al., 2017[4]). TNF are cell signaling proteins (cytokines) involved in systemic inflammation that make up the acute phase reaction caused by local or systemic disturbances to the homeostasis of the body.

### Haptoglobin

Haptoglobin (Hp) is an acute phase protein that is also known as a hemoglobin (Hb)-binding serum protein. It is found in human serum at a normal level of 0-300 mg/dL and and is mainly synthesized in the liver (Katnik and Jadach, 1996[40]). Hp serum levels are known to increase in response to injury as well as in the acute phase by as much as 3- to 8-folds (Dobryszycka, 1997[20]). IL-6, IL-1 and TNF-α are shown to induce the production of hepatic Hp, which is also present in some non-hepatic cells such as adipocytes and lung cells. Increased levels of Hp from both hepatic and non-hepatic sources are observed after inflammation (Asleh and Levy, 2005[5]). In addition, unmitigated stress and nitric oxide can cause changes in the blood flow, which in turn encourages the arteries to induce the expression of Hp thus, further influencing IL-6 expression. Furthermore, Hp from arteries are implicated in cell migration and the reconstruction of arteries (Smeets et al., 2002[71]). 

The Hb released during hemolysis acts as a potent oxidant whereby the progression of atherosclerosis is enhanced due to the entry of Hb into the walls of vessels thereby mediating lipoprotein oxidation. Hb-induced oxidative damage can be obstructed via the antioxidant exerting property of Hp. In addition, Hp utilizes CD163 macrophage scavenger receptor in facilitating the removal of Hb. In humans, Hp consists of two common alleles (denoted as 1 and 2) which combines to form genotypes (Hp1-1, Hp2-1 and Hp2-2) with three possibilities. Asleh and Levy (2005[5]) recently showed that the Hp genotype represents a risk factor for the contraction of diabetic vascular complications. Precisely, they determined that diabetics with the Hp 2-2 genotype have a higher chance of contracting nephropathy, retinopathy and cardiovascular diseases in comparison to those individuals with the Hp2-1 or Hp1-1 genotypes.

### Zinc-alpha-2-glycoprotein (ZAG)

Zinc alpha-2-glycoprotein (ZAG) is a 40 kDa single chain polypeptide, secreted in various body fluids (Hassan et al., 2008[37]). After the discovery of this molecule, many researches have vastly documented both the structure and function of this protein. However, in spite of all the research, the function of ZAG is still unknown. ZAG is present in a variety of epithelia and is secreted into many body fluids (Wang et al., 2016[83]). Although the function of ZAG is ambiguous, a high degree of similarity with classic MHC-I molecules suggest its role in immunomodulation (McDermott et al., 2006[55]). 

ZAG was reported to be associated with a wide variety of diseases and cellular disorders including obesity, prostate and bladder cancers, cachexia, as well as cell proliferation processes (Wang et al., 2016[83]). Immunohistochemical analysis has shown that ZAG is expressed mainly in the tubules of the human kidneys. Similarly, proteomic analysis revealed that urinary level of ZAG increased corresponding to patients with diabetes and this correlation might be useful in the process of applying ZAG as a potential biomarker of DN (Jain et al., 2005[39]). Besides ZAG, there are other glycoproteins that have been revealed as being associated with diabetic patients. Jain et al. (2005[39]) demonstrated the use of 2DGE in combination with Matrix Assisted Laser Desorption Ionization Time of Flight (MALDI-TOF) mass spectrometry and western blot for the identification of the group of glycoproteins that responds to high blood glucose conditions such as ZAG, α-1 acid glycoprotein, α-1 microglobulin and IgG. These findings are in accordance with our discovery (Table 1(Tab. 1)).

## Utilization of Bioinformatics in Diabetes Research

Bioinformatics is an interdisciplinary field of study encompassing the use of computer science, biology, chemistry, mathematics and engineering for analysing and interpreting biological information (Spengler, 2000[72]). 

Currently, major trends in this field include ligand-based drug design for modulating metabolic pathways and structure-based drug design (protein structure, molecular docking, molecular dynamics, etc.) for studying the impact of mutations on protein folding, stability and function. 

In recent years, the advancement of technology and their integration in science has led to the use of bioinformatics tools for the prediction and analysis of the rapidly increasing proteomics data. As part of an interconnected large networks, the expression profiles of proteins and peptides can be modulated in diseases. Furthermore, the process of identifying novel biomarkers for DM diagnosis can make simultaneous use of multiple, heterogeneous data sets in contrast to examining alone. This approach may be useful in highlighting the knowledge pertaining to the progression and pathophysiology of DN. 

Presently in the literature, the focus of bioinformatics as applied to DM is mainly concerned with using microarray data (Baelde et al., 2004[6]; Fujita et al., 2006[27]; Lamb et al., 2006[44]) for linking genes with diseases as well as using miRNA data (Eissa et al., 2016[23]; Wu et al., 2014[88]) for determining novel biomarkers of DN. In addition, the vast data pandemonium has brought about the need for big data analysis which has further paved way for many urinary proteomics and bioinformatics research conducted in recent years (Maahs et al., 2010[51]; Meier et al., 2005[56]; Varemo et al., 2015[79]; Zhang et al., 2015[92]). 

**Figure 3:**
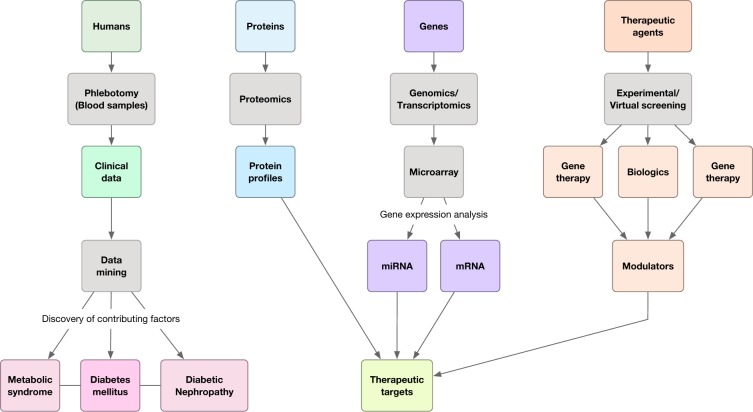
Conceptual framework for the utilization of bioinformatics and omics for studying DM and its complications.

Herein, we summarize these efforts in utilizing molecular data for computational rationalization of proteomics data in DN (Figure 3(Fig. 3)). Despite several genome-wide association studies for investigating common and unique genetic variants from exome sequencing, the genetic architecture of DN still remains poorly understood (Bonomo et al., 2014[9]; Pezzolesi et al., 2009[64]; Sandholm et al., 2012[69]). In that regard, proteome- and transcriptome-driven studies have been utilized in order to discover novel biomarkers for diabetes (Varemo et al., 2015[79]; Marinkovic and Oresic, 2016[53]). Meier et al. (2005[56]) investigated the urinary proteome profiles of healthy adolescents with T1D in comparison to controls. Thus, this study sets the stage for other studies by demonstrating that differences in urinary proteome profiles could be detected at the onset of diabetic kidney disease. 

In the largest urinary peptidome study of diabetes to date, Maahs et al. (2010[51]) observed lower urinary levels of collagen and uromodulin in diabetic cases with normal renal function as compared to that of controls. Furthermore, Zhang et al. (2015[92]) used bead-based matrix-assisted laser desorption ionization time-of-flight mass spectrometry (MALDI-TOF MS), which allows the enrichment and analysis of small proteins, to discover two urinary proteomic fragments of fibrinogen α-chain and prothrombin. These fragments are important, closely related players in blood coagulation where the lack of which have been linked to a high risk of thrombosis in patients with T2D. 

Additionally, a number of computational studies have also been conducted on the protein-ligand interactions of RBP in the context of DM diagnosis and therapy (Motani et al., 2009[59]; Naylor and Newcomer, 1999[62]; Torabi et al., 2017[75]). A number of proteomic and peptidomic analyses of clinical samples including plasma and urine from diabetic patients have been reported as biomarkers that predict the progression of nephropathy. Varieties in protein expression profiles are present according to the type of cell, tissue, organ, or specimen, which can shed light on the location of damage. However, multiple data sets of potential biological markers can be extremely useful when considered together. Van et al. (2017[77]) investigated the biological implications of differentially excreted urinary proteins in patients with DN by taking advantage of published, relevant data coupled with protein-protein interactions (PPI) network analysis. Artificially constructed PPI networks were applied for comprehensively identifying common and stage-specific biological processes in diabetic kidney disease by including all proteins biomarkers associated with particular stages of the disease. Therefore, in the above mentioned study, the author used candidate biomarkers with differential excretion between cases and controls extracted from 31 samples and equally weighted as input data. 

The most promising DN urinary biomarkers in the various nephrons were elucidated. Data from the Human Protein Atlas was used to determine the differences in protein expressions induced in renal tissues as compared to their normal expressions (Uhlen et al., 2010[76]) and the existing literature. Comparison of enriched biological processes in uncomplicated diabetes and incipient DN indicates its involvement in the regulation of wound healing (i.e the umbrella term used for describing complex biological processes involved in the body's response to injury and subsequent repair), coagulation, inflammation, cholesterol and lipid metabolism, as well as stress responses. Example of inflammatory factors present includes several acute phase reactant proteins, α1-antitrypsin, haptoglobin, fibrinogen, and transferrin in uncomplicated diabetes and incipient DN or borderline hyperglycemic patients has been reported (Long et al., 2016[50]; Van et al., 2017[77]). 

In the case of overt DN patients, the aforementioned biological processes are also involved just as in the case of incipient DN, whereby elevated protein biomarkers corresponding to coagulation, inflammation and stress responses are determined. Retinol-binding protein 4 (RBP4), Transthyretin (TTR), β2-microglobulin (B2M), α2-glycoprotein are revealed to be the most responsive at this stage.

Inflammation can be linked to the pathway of wound healing in the same way as coagulation and extracellular matrix regulation as found in uncomplicated diabetes. Macrophages infiltrate injury sites during the inflammation process to clear cellular debris via phagocytosis and thereby encouraging the migration of other cells of the innate immunity (Gurtner et al., 2008[34]). Thus, in order for the injured tissue to heal, the inflammation process has to eventually be down-regulated. However, the inflammatory process is sustained by chronic hyperglycemia therefore predisposing tissues to progressive diabetic kidney disease (Navarro-Gonzalez and Mora-Fernandez, 2008[61]). Taken together, these correlations reveal the success of artificially constructed biological process networks in identifying promising biomarkers for each stage of DN. Many studies have supported the use of PPI as a basis for determining novel connections between proteins involved in the progression of diabetes (Abedi and Gheisari, 2015[1]; Saito et al., 2016[68]; Varemo et al., 2015[79]).

Moreover, various data mining techniques have been successfully utilized in DM research as to discover hidden patterns and relationships (Worachartcheewan et al., 2010[84], 2013[85][86], 2015[87]). Fang (2009[25]) used data mining techniques such as clustering, classification and regression models for the identification of diabetic patients of a large health care enterprise. Bagherzadeh-Khiabani et al. (2016[7]) made use of a clinical data set comprising of 803 pre-diabetic females and compared several common feature selection algorithms to predict the likelihood of DM. In another work, Georga et al. (2015[29]) applied random forest (Breiman, 2001[10]) and RReliefF (Robnik-Šikonja and Kononenko, 2003[67]) to evaluate a number of features, with respect to their ability to predict the short term subcutaneous glucose concentrations in diabetic patients. 

In addition, many complications of DM have been studied using machine learning approaches and data mining techniques. In a more general aspect, Lagani et al. (2015[43]) targeted several diabetic complications, such as cardiovascular diseases (CVD), hypoglycemia, proteinuria, ketoacidosis, microalbuminuria, neuropathy, and retinopathy in an effort to identify the smallest set of clinical parameters affording the best predictive accuracy that makes use of the aforementioned diabetic complications as parameters. 

In the case of nephropathy, Huang et al. (2015[38]) employed a decision tree-based prediction tool that combines both genetic and clinical features in order to identify DN in patients with T2D. On the other hand, Leung et al. (2013[46]) compared the current age of patients, age at diagnosis, systolic blood pressure using numerous machine learning methods (e.g. partial least square regression, classification and regression tree, random forest, Naïve Bayes, neural networks and support vector machine) through which genetic polymorphisms of the uteroglobin and lipid metabolism arose as the most efficient predictors. Cho et al. (2008[16]) applied machine learning for predicting the onset of DN via risk factor analysis in which patients with high microalbumin levels are considered to be at high risk for DN. Similarly, DuBrava et al. (2017[22]) made use of the random forest learning algorithm (Breiman, 2001[10]) for shedding light on factors contributing the likelihood of acquiring diabetic peripheral neuropathy (DPN). In addition, Shoombuatong et al. (2015[70]) performed the first large-scale study for investigating the chemical space of potential anti-diabetic agents targeting dipeptidyl peptidase-4.

The potential benefits (while also taking into account possible risk factors) of early DM detection includes the following: im-proved quality of life, longevity, reduction of severity and frequency of disease, prevention and delay of its complications as well as reduction of health care costs. In this context, data mining and machine learning are key mediators that can help provide insight into possible relationships among molecules and conditions such as gene-gene, protein-protein, drug-drug, drug-disease or gene-disease interactions.

## Conclusion

The increasing worldwide prevalence of DM is in parallel with the rise of obesity. As previously mentioned, the global statistics as of 2015 estimated that 415 million people are currently living with diabetes with an expected rise of up to 642 million by 2040 according to the International Diabetes Federation (Ogurtsova et al., 2017[63]). These statistics provide a grim outlook. DN is the leading cause of kidney disease and major complications in diabetic patients. It affects about 40 % of patients with type-1 and type-2 diabetes. In addition, DN continues to account for a large proportion of CKD and remains by far the most common cause of chronic dialysis leading to the escalation of health care costs. 

Although, DN does not always progress from one stage to the next, the presence of microalbuminuria is widely accepted as the first clinical sign of this disease complication. In addition, prior studies indicated that increased levels of albuminuria is not a sensitive biomarker for DN determination owing to the late detection of its levels whereby the kidneys of some diabetic patients had already sustained glomerular and tubulointerstitial damage by the time that increased levels are detected. Therefore, there is an urgent need to discover new, early biomarkers for the detection of DN. Herein, we have demonstrated various biomarkers and have also revealed the pathophysiological roles of hyperglycemia-related renal diseases.

Proteomics is now beginning to deliver large information for several diseases including diabetes, neuropathy, kidney disease, DN and other vascular complications. Currently, single biomarkers are used in the detection of several diseases but may need to be replaced with multiple biomarkers due to their higher specificity and accuracy. Furthermore, systemic biological approaches are based not only on proteomics but also on other "omics" areas such as metabolomics or transcriptomics and have served as a huge avenue for data in the understanding of diseases at the molecular level. In addition, the identification of promising therapeutic targets for the improvement of patient care is an active area of research. However, there is a lack of clear consensus on the definite explanation of reliable biomarkers, the required performance of such multiple biomarkers, the type of studies needed to obtain results that are acceptable and sufficient for clinical implementation and whether the assessed biomarkers should be implemented as a desirable therapeutic avenue. Guidelines for the efficient use of biomarkers in specific disease conditions could be beneficial in their correct and lasting implementation. Taken together, all supportive data indicate that the onset of DN does not depend on few particular features, but instead depends on a complex interrelationship involving many features. Thus, the integration of sufficient bioinformatics tools might be the key for improving data implementation in order to gain highly accurate information and discover specific novel biomarkers for DN and other diabetes complications.

## Acknowledgements

This work is supported by Annual Research Budgets of Mahidol University (B.E. 2556-2558 and B.E. 2557-2559).

## Figures and Tables

**Table 1:**
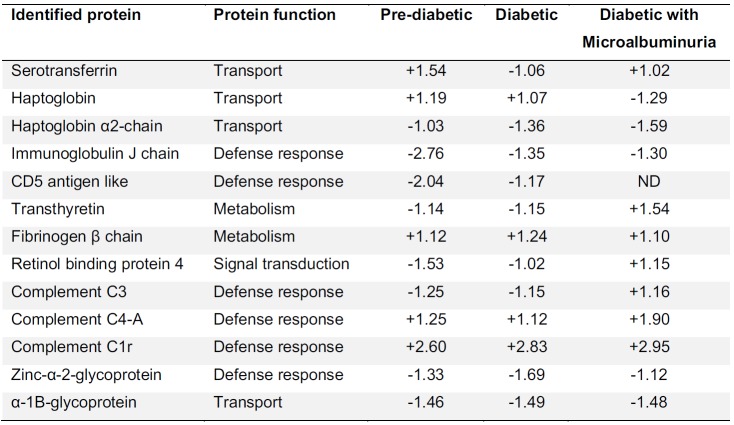
Comparison of plasma protein differential expression in hyperglycemic (glucose > 126 mg/dL) and normal conditions (glucose < 100 mg/dl)
